# Microinjection manipulation decreases the expression of GABA‐A receptor signaling pathway genes in mouse embryos derived using intracytoplasmic sperm injection

**DOI:** 10.1002/jcla.23584

**Published:** 2020-09-20

**Authors:** Zili Zhang, Ting Wang, Juanhua Huang, Yonghan Huang, Qingxue Zhang

**Affiliations:** ^1^ Department of Reproductive Medicine Center Sun Yat‐Sen Memorial Hospital, Sun Yat‐Sen University Guangzhou China; ^2^ Department of Reproductive Medicine Center The First People’s Hospital of Foshan (Affiliated FoShan Hospital of Sun Yat‐sen University) Foshan China

**Keywords:** embryonic development, GABA‐A receptor signaling pathway, gene expression, intracytoplasmic sperm injection, microinjection

## Abstract

**Background:**

The GABA‐A receptor signaling pathway regulates proliferation, differentiation, apoptosis, and responses to overt DNA damage during embryonic development.

**Methods:**

To analyze the gene expression after intracytoplasmic sperm injection (ICSI) and in in vivo mouse embryos, the global pattern of gene expression dataset, GSE23009, was obtained from the Gene Expression Omnibus database. Genes with differential expression were identified using the R software package, and RT‐qPCR was performed to confirm the microarray results.

**Results:**

Mouse blastocysts derived from ICSI fertilization had decreased expression of GABA‐A receptor signaling pathway genes. However, the mechanisms underlying these changes were not elucidated. The gene expression of the GABA‐A pathway was not significantly different between blastocysts obtained from IVF and in vivo fertilization. However, microinjection after IVF significantly reduced the expression of the GABA‐A pathway gene to levels similar to those in the ICSI group.

**Conclusion:**

Based on our results, decreased gene expression is a result of the microinjection manipulation performed during ICSI.

## Graphical abstract

(A) The red points represent genes that were upregulated based on a |fold change|> 1.0 and a corrected *P*‐value of < .05. The blue points represent downregulated genes screened based on a |fold change| < 1.0 and a corrected *P*‐value of < .05. The black points represent genes with no significant differences in expression. (B) The GO analysis divided DEGs into three functional groups, including molecular function, biological processes, and cellular components.

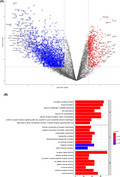

## 1 INTRODUCTION

Intracytoplasmic sperm injection (ICSI) has been extensively applied to patients in the clinic to overcome the most severe types of male infertility.^1^, ^2^ Tens of thousands of infants have been born worldwide due to the use of ICSI.^3^ However, ICSI skips several physiological steps that occur during natural fertilization; therefore, there is a widespread concern regarding whether ICSI induces developmental defects in the offspring produced using this method. Based on emerging evidence, infants conceived via in vitro fertilization (IVF) or ICSI are at a higher risk of developing birth defects than those who are naturally conceived.^4^ After the systematic analysis of long‐term follow‐up data, Catford et al suggested that infants conceived via ICSI are at a higher risk of having poor physical health, as identified by metabolic and reproductive endpoints.^5^

One pathway that may underlie the negative effects in children conceived using ICSI is the GABA pathway. GABA is a major suppressive neurotransmitter in the mammalian central nervous system that is synthesized from glutamate via L‐glutamic acid decarboxylase. GAT, a GABA transporter, is present in the plasma membrane of glial cells and nerve terminals. It plays a critical role in the termination of synaptic transmission. GABA enters the cell via GAT and is then converted to gamma‐hydroxybutyrate or succinate; the latter eventually enters the citric acid cycle. Three GABA receptors have been identified: GABA‐A, ‐B, and ‐C. GABA‐A and ‐C receptors are transmitter‐gated ion channels, whereas GABA‐B receptor is a G protein‐coupled receptor that is activated by baclofen. GABA‐A receptors are heterooligomeric Cl^−^ channels that are modulated by barbiturates and benzodiazepines. Evidence suggests that the activity of the GABA receptor pathway decreases after ICSI.^6^ A study has shown that mouse embryos lacking the GABA‐A receptor display a severe phenotype and exhibit abnormal growth and cell proliferation as well as attenuation of neural development.^7^

In the present study, we assessed the gene expression dataset GSE23009 from the Gene Expression Omnibus (GEO) database. We then validated these results using RT‐qPCR to identify changes in gene expression in the components of the GABA‐A receptor pathway in embryos derived using in vivo fertilization, IVF, and ICSI. By comparing the gene expression between the in vivo fertilization and IVF groups from the GSE23009 dataset, we identified that in vitro culture does not affect the expression of these genes. Moreover, treatment with hyaluronidase did not affect the gene expression of the factors in this signaling pathway. However, microinjection after IVF significantly reduced the expression of GABA‐A receptor pathway genes. Our findings suggest that microinjection manipulation during ICSI leads to a decrease in the expression of the GABA‐A receptor pathway genes, whereas in vivo fertilization and IVF do not decrease the expression.

## 2 MATERIALS AND METHODS

### 2.1 Gene expression data and identification of differentially expressed genes (DEGs)

The gene expression dataset GSE23009 was obtained from the GEO database. The GEO dataset platform used was GPL1261 Mouse Genome 430 2.0 Array (Affymetrix, Santa Clara, CA, USA). Differential expression was analyzed using the limma R package. Biological term classification and enrichment analysis were performed using the clusterProfiler R package.^8^ Limma and clusterProfiler R packages were obtained from the Bioconductor project. R version 3.3.3 was used (Auckland, New Zealand). A |logFC|> 1, a *P*‐value < .05, and an adjusted *P*‐value < .05 indicated a statistically significant difference for DEGs.

### 2.2 collection of mature oocytes and spermatozoa

MII oocytes were collected from 6‐ to 10‐week‐old C57BL/6NHsd mice. Mice were treated with pregnant mare serum gonadotropin (5 IU, PMSG; Sigma, St. Louis, MO) and human chorionic gonadotropin (5 IU, HCG; Sigma). Female mice were manually restrained for intraperitoneal (IP) injections. HCG was IP injected into female mice 47‐49 h after their last PMSG injection. Approximately 16 h after HCG injection, oviducts were collected from female mice and placed in M2 media (Sigma). Each oviduct was then transferred to a dish containing M2 media and hyaluronidase (Sigma). The ampulla was incised and washed with M2 media to obtain the oocytes. Oocytes were collected and placed into a 100‐µL drop of KSOM (Sigma) using embryo‐tested mineral oil (Sigma) that had been equilibrated at 37°C in a 5% CO_2_ atmosphere. When required, cumulus cells were treated with bovine testes hyaluronidase (0.1%, Sigma). Mouse sperm was harvested from the cauda epididymis of 10‐15‐week‐old C57BL/6NHsd male mice, and the spermatozoa were suspended in M2 medium for 20 min. All animal experiments and procedures were approved by the Institutional Animal Care and Use Committee of Sun Yat‐Sen Memorial Hospital, Sun Yat‐Sen University.

### 2.3 ICSI procedure

ICSI was performed using a previously described method.^9^ A drop of the sperm suspension was added to 12% (w/v) polyvinyl pyrrolidone (PVP, MW 360 kDa; Sigma) in M2 media. The head of a single spermatozoon was separated from the tail using a TPC pipette (TPC, Australia) and was subsequently injected into the oocyte. Spermatozoa‐injected oocytes were cultivated in KSOM medium for 24 h under damp conditions with 5% O_2_ and 5% CO_2_ at 37°C. The two‐cell stage embryos were then cultivated for 2 more days to obtain blastocyst‐stage embryos.

### 2.4 RNA extraction and cDNA preparation

Total RNA was extracted and cDNA was synthesized as described previously.^10^ RNA was isolated using the RNAeasy Micro Kit (Qiagen) according to the manufacturer's instructions. RNA concentration and quality were measured using a NanoDrop spectrophotometer (Thermo). cDNA templates were synthesized from RNA (2 μg) using a reverse transcription kit (Beyotime).

### 2.5 Gene expression analysis via qPCR

qPCR was performed using a RT‐PCR system (Roche) with the SYBR Green Master mix (Roche). Each reaction was performed in a volume of 10 μL with 50 ng of cDNA template. All samples were analyzed three times and the reactions were performed for 40 cycles. Primers for the following genes were designed using perlPrimer software: *Gabra1*, *Gabra6*, *Gabrg1*, *Gabrg2*, *Gabbr1*, and *Gapdh*. Gene expression was standardized to the reference gene (in vivo/IVF) and calculated using the ΔΔCT method.

### 2.6 Data analysis

The data were analyzed using the R software and appropriate Bioconductor packages. Comparisons of qPCR data between the two groups were conducted using the two‐tailed t test using the GraphPad Prism software^6^ (San Diego, CA). A *P*‐value of < .05 was considered statistically significant.

## 3 RESULTS

### 3.1 Microarray data validation via RT‐qPCR

**Figure 1:**
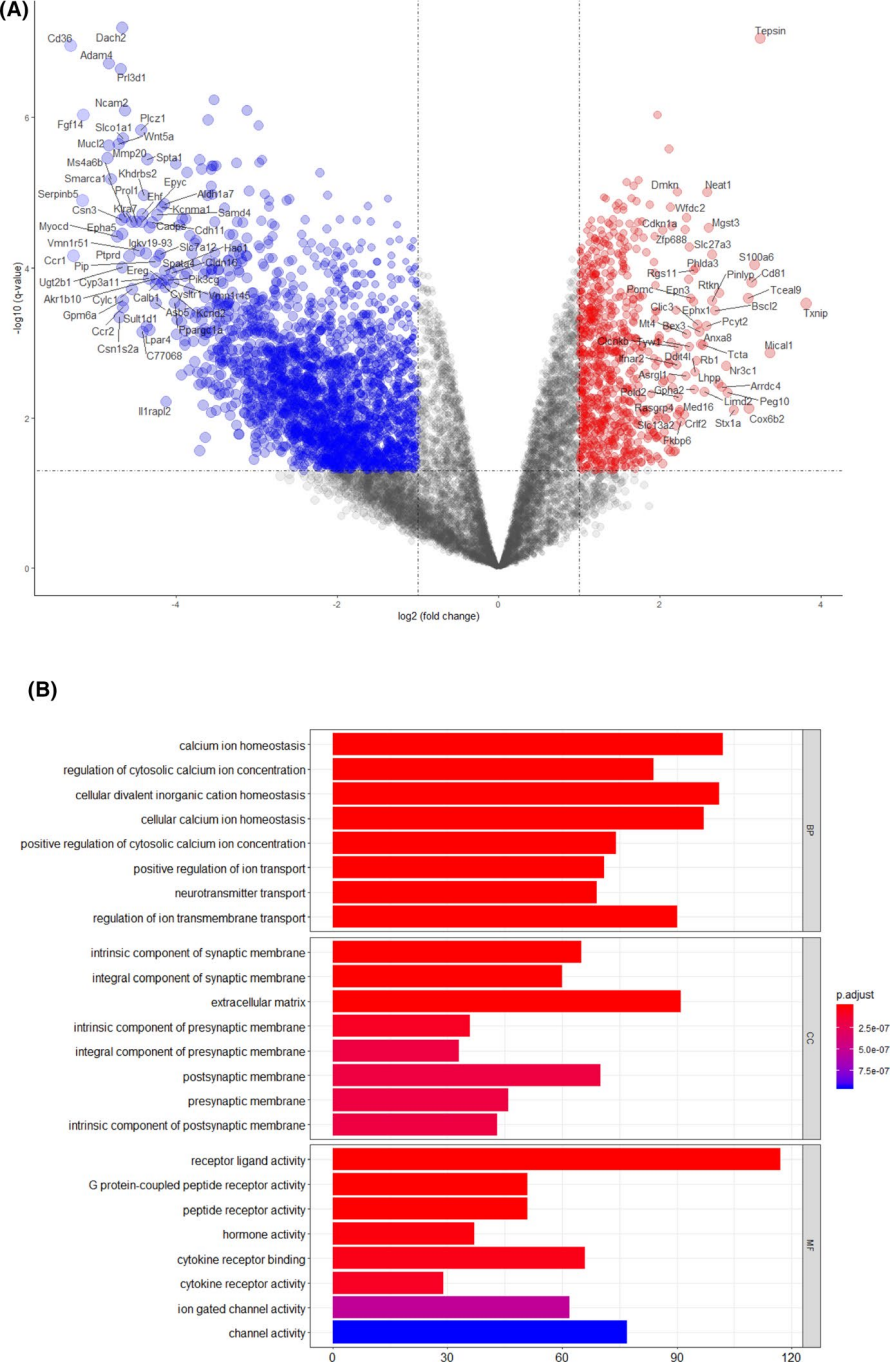
Differentially expressed genes between the in vivo and ICSI groups. (A) Red points represent genes that were upregulated based on |fold change|> 1.0 and corrected *P*‐value of < .05. Blue points represent genes that were downregulated based on |fold change| < 1.0 and corrected *P*‐value of < .05. Black points represent genes with no significant differences in expression. (B) GO analysis divided the DEGs into three functional groups based on molecular function, biological processes, and cellular components

The mouse Affymetrix 430 2.0 microarray dataset GSE23009 was standardized, and the data from the in vivo and ICSI groups were extracted and screened using the limma package of the R software (adjusted *P*‐value < .05, |logFC|> 1). From this dataset, 2,270 DEGs were obtained, including 1,593 genes that were downregulated and 677 genes that were upregulated (Figure 1A). GO pathway enrichment analysis was conducted to identify the potential biological functions of 2,270 DEGs. These genes were significantly enriched in multiple biological processes related to the cell membrane, including calcium ion homeostasis, cellular divalent inorganic cation homeostasis, and regulation of ion transmembrane transport (Figure 1B). In addition, the differences in gene expression related to the GABA receptor pathway between the in vivo and ICSI groups were analyzed. As shown in Table 1, the expression of these genes was dramatically decreased in the ICSI group.

**Table 1:** Comparison of the differentially expressed genes of the GABA receptor signaling pathway between the ICSI and in vivo groups from the GSE23009 dataset

ICSI vs. in vivo GABA receptor signaling genes		
SYMBOL	logFC	*P* ‐value
Abat	1.536886225	.070525
Gabra1	−2.347018069	.000314
Gabra2	−1.64489691	.041906
Gabra3	−1.920377457	.023715
Gabra6	−3.09380697	.001948
Gabrb1	−1.400364478	.024582
Gabrb2	−1.445795787	.00387
Gabrb3	−2.235034449	.016888
Gabrg1	−2.832220777	.001598
Gabrg2	−3.570115231	4.87E‐06
Gabrg3	−1.499243177	.052093
Gabrd	−1.158367378	.009197
Gabre	−2.360656244	.014666
Gabrp	−1.180994294	.024538
Gabrq	−1.757867336	.01424
Gabrr1	−1.806127276	.021756
Gabrr2	−0.756998697	.422016
Gabbr1	−0.104550547	.643326
Gad1	−1.838111094	.004676
Gad2	−1.742447122	.002498
Aldh9a1	1.247019756	.007693

**Figure 2:**
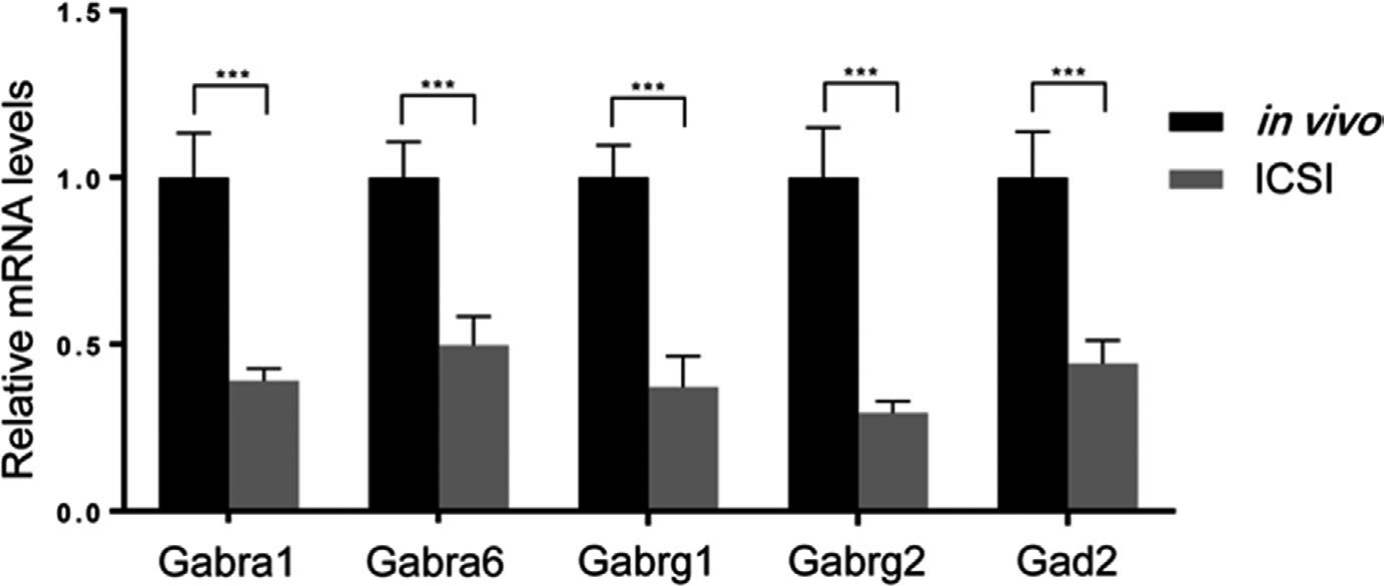
Validation of the microarray data using RT‐qPCR. (A) qPCR analysis of the expressions of Gabra1, Gabra6, Gabrg1, Gabrg2, and Gad2 in blastocysts derived from in vivo and ICSI fertilization (means ± SEM; *** *P* < .001; n ≥ 20)

To identify the expression of GABA receptor pathway genes as well as to validate the microarray data, we extracted total RNA from expanded blastocysts displaying similar morphology (n ≥ 20) from mice. We chose five genes with the lowest *P*‐values in Table 1 to compare the gene expression of the components of the GABA receptor pathway between in vivo and ICSI blastocysts using RT‐qPCR (Figure 2). The results showed a similar trend between the qPCR and microarray data, validating that there is substantial downregulation of the genes of the GABA‐A receptor signaling pathway after ICSI.

### 3.2 Effects of in vitro culture and hyaluronidase treatment on the expression of GABA signaling pathway genes in embryos

In contrast to natural intrauterine conception and growth, embryos obtained from ICSI and IVF were cultured in vitro. We analyzed the in vivo and IVF groups using the R software to determine whether in vitro culture affects the expression of GABA receptor pathway genes (Table S1). A statistically significant difference was not observed between the in vivo and IVF groups. These findings suggest that in vitro culture does not alter the expression of GABA receptor pathway genes.

On the other hand, ICSI is more aggressive than traditional IVF because oocytes from ICSI are treated with hyaluronidase during the process of denuding and are damaged by microinjection.^11^ We compared the expression of GABA signaling pathway genes between embryos obtained from IVF with the denuding process and conventional IVF. Oocytes were randomly assigned to a treatment group, group with the removal of the cumulus (N = 22 oocytes), or the conventional IVF group (control; N = 31 oocytes). The cumulus was removed immediately after oocyte retrieval and insemination was performed after 6‐8 h. RNA was subsequently isolated from the blastocysts (n ≥ 20), and RT‐qPCR was used to evaluate gene expression. Significant differences were not detected between the blastocysts from the treated and conventional IVF control groups (Figure S1), suggesting that reduced expression of GABA receptor pathway genes is not as a result of hyaluronidase treatment.

### 3.3 Microinjection during ICSI decreased the expression of GABA‐A receptor pathway genes

**Figure 3:**
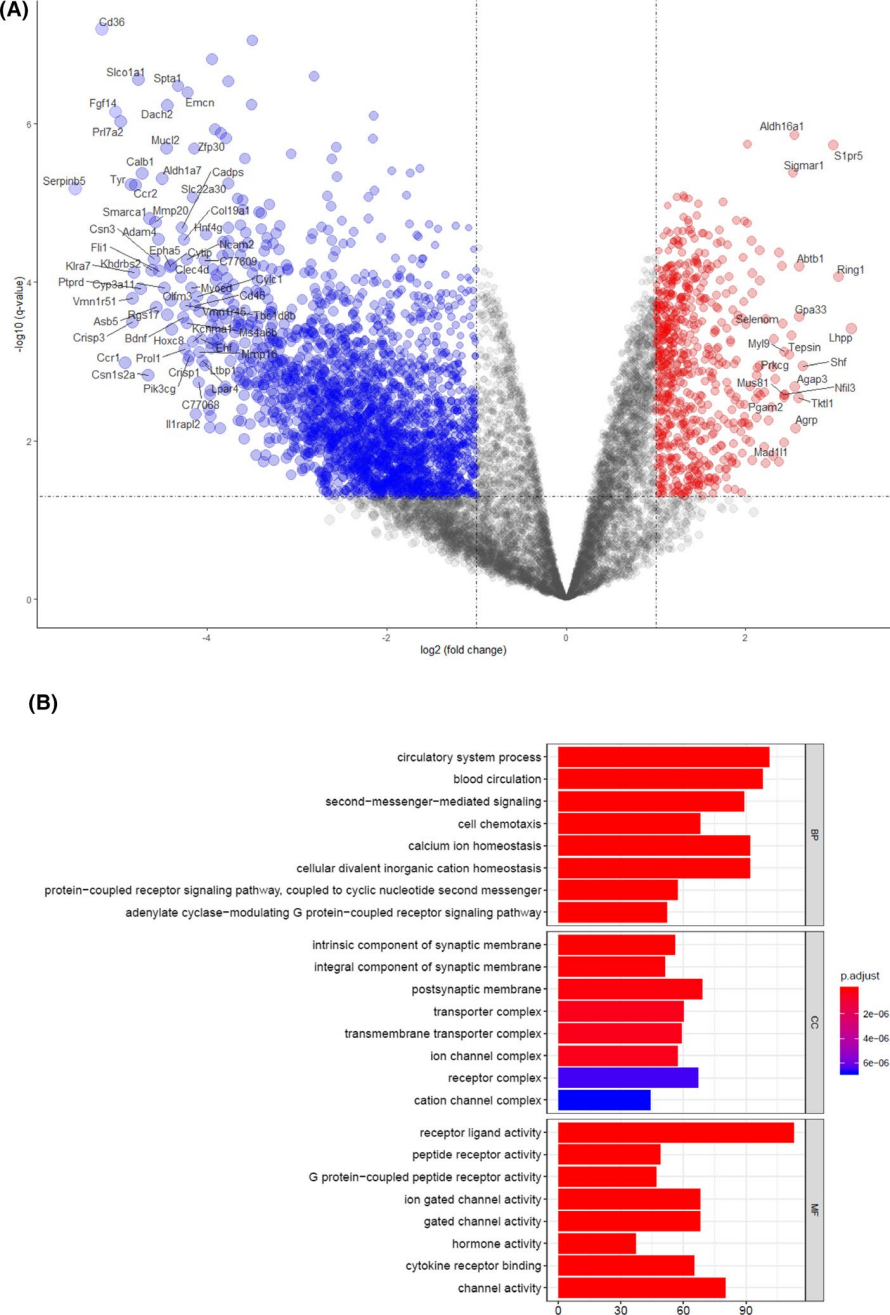
Differentially expressed genes between the IVF and ICSI groups. (A) Red points represent genes that were upregulated based on |fold change|> 1.0 and corrected *P*‐value of < .05. Blue points represent downregulated genes screened based on |fold change| < 1.0 and corrected *P*‐value of < .05. Black points represent genes with no significant differences in expression. (B) GO analysis divided DEGs into three functional groups based on molecular function, biological processes, and cellular components

**Figure 4:**
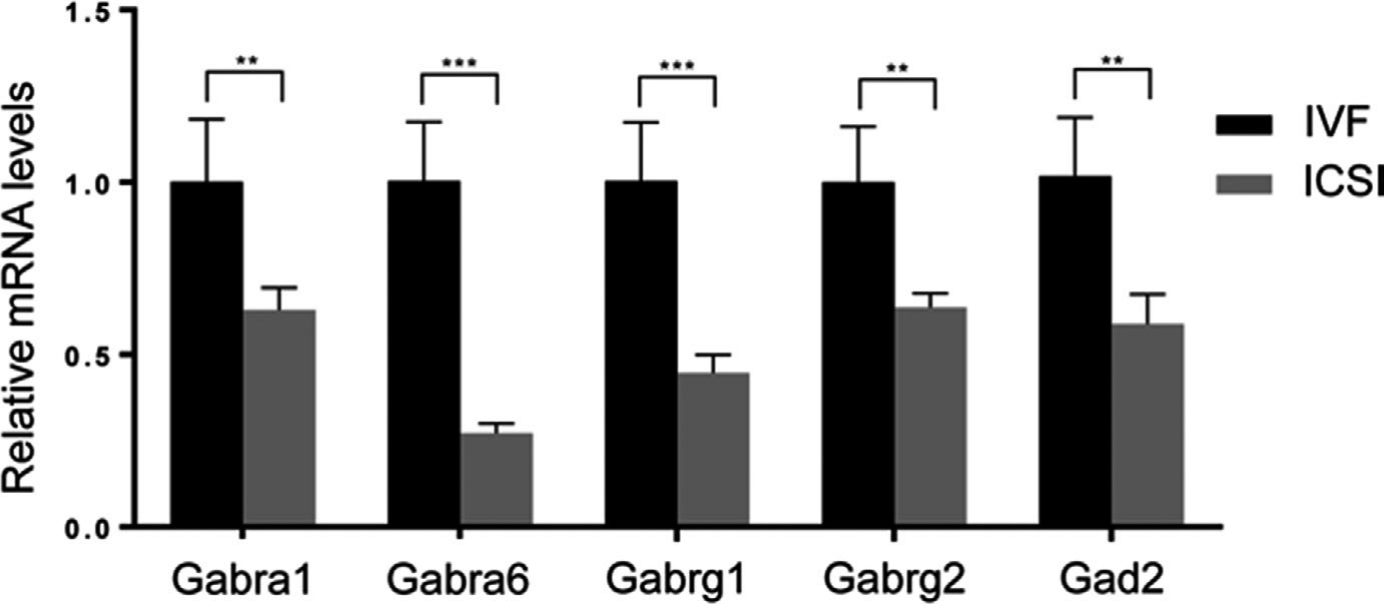
Differentially expressed genes of the GABA‐A pathway between the IVF and ICSI groups. (A) qPCR analysis of the expression of *Gabra1*, *Gabra6*, *Gabrg1*, *Gabrg2*, and *Gad2* in blastocysts derived from IVF and ICSI fertilization (means ± SEM; *** *P* < .001 and ** *P* < .01; n ≥ 20)

Previous studies have shown that the site and technique used to rupture the oolemma exert substantial effects on fertilization and the rate of damage during ICSI 12. We compared the DEGs between the ICSI and IVF groups from dataset GSE23009 to specifically evaluate the effect of microinjection during ICSI. Notably, 2,156 DEGs were identified, including 1,550 genes that were downregulated and 606 genes that were upregulated (Figure 3A). GO pathway enrichment analysis was conducted to identify the potential biological functions of the 2,156 DEGs. DEGs were significantly enriched in multiple biological processes (Figure 3B). As expected, the expression of GABA‐A pathway genes was significantly decreased in the ICSI group (Table 2). qPCR was used to validate the microarray data; the qPCR results were consistent with the microarray data (Figure 4A). Therefore, microinjection may be the main reason for the decreased gene expression of the components of the GABA‐A receptor pathway.

**Table 2:** Comparison of the differentially expressed genes of the GABA receptor signaling pathway between the ICSI and IVF groups from the GSE23009 dataset

ICSI vs IVF GABA receptor signaling genes		
SYMBOL	logFC	*P* ‐value
Abat	1.285286	.000561
Gabra1	−1.6891	.015155
Gabra2	−0.66945	.278119
Gabra3	−1.28304	.177927
Gabra6	−3.09727	.014802
Gabrb1	−2.16039	.007019
Gabrb2	−1.72421	.000914
Gabrb3	−2.7325	.034138
Gabrg1	−2.57725	.011375
Gabrg2	−2.18793	.004347
Gabrg3	−0.37283	.567334
Gabrd	−0.68904	.025514
Gabre	−2.62815	.000447
Gabrp	−0.17934	.835021
Gabrq	−1.56763	.098018
Gabrr1	−2.82486	.002122
Gabrr2	−0.94865	.189757
Gabbr1	−0.02888	.909559
Gad1	−1.43608	.017436
Gad2	−1.14217	.042478
Aldh9a1	0.579371	.063666

### 3.4 Microinjection damaged the Oolemma and decreased the expression of GABA‐A receptor pathway genes

**Figure 5:**
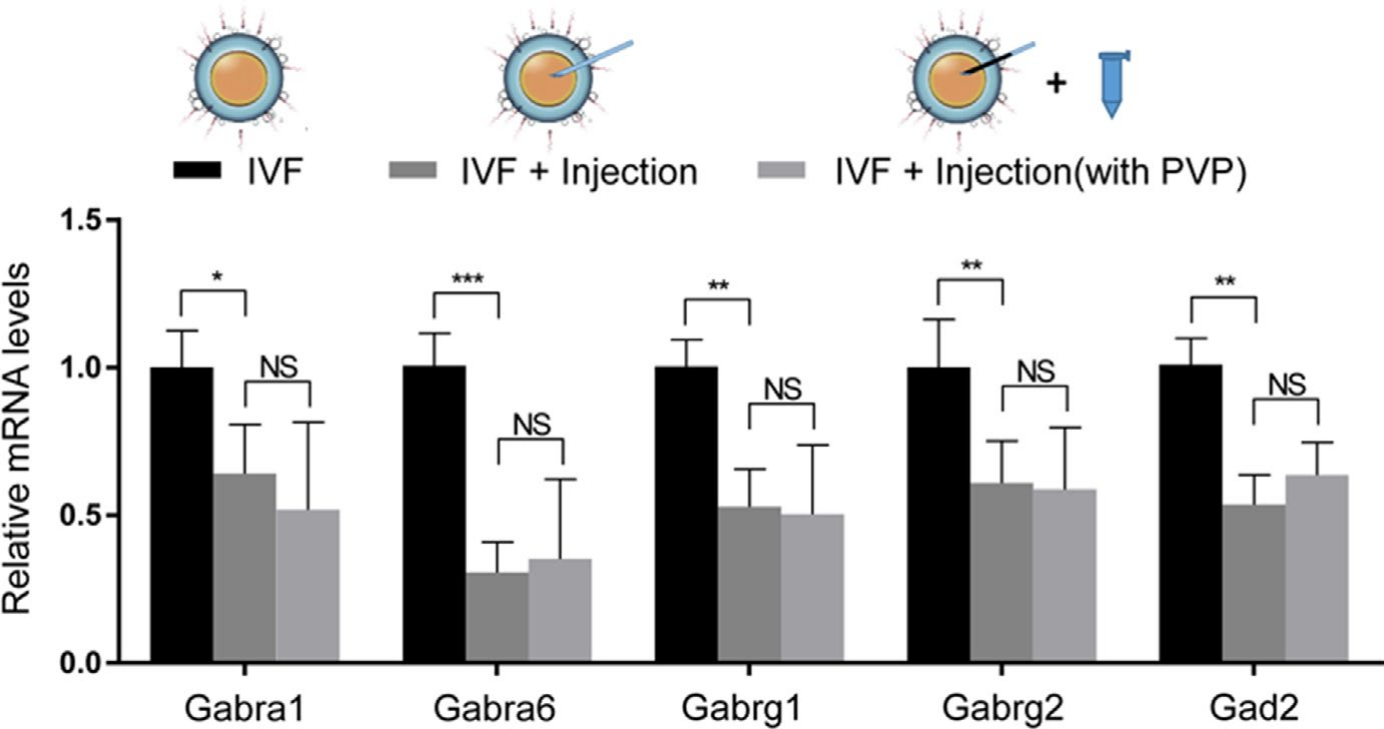
Microinjection during ICSI decreased the expression of genes of the GABA‐A pathway. (A) qPCR analysis of the expression of *Gabra1*, *Gabra6*, *Gabrg1*, *Gabrg2*, and *Gad2* in embryos derived from IVF fertilization after injection with KSOM or PVP (means ± SEM; ***P < .001, **P < .01, and *P < .05; ns, not significant; n ≥ 20)

Survival, fertilization, and pregnancy rates are sensitive to the damage caused by microinjection manipulation during ICSI,^13^, ^14^ suggesting that GABA‐A receptors on the oolemma may be injured during this process. Therefore, microinjection was performed in the embryos after IVF. The effect of PVP was also evaluated (Figure 5A). PVP appeared to exert a slight effect on the gene expression of GABA‐A pathway. However, the gene expression of factors in the GABA‐A receptor pathway was significantly decreased after microinjection manipulation, suggesting that microinjection damages the GABA‐A receptors on the oolemma and subsequently alters their gene expression. In summary, the decreased gene expression of the GABA‐A receptor pathway results from microinjection during ICSI.

## 4 DISCUSSION

We analyzed the microarray dataset GSE23009 to investigate the reason for the reduced expression of GABA‐A receptor pathway genes during ICSI. The gene expression of the ICSI group was dramatically decreased, while those of the IVF group were generally unaffected. The decreased gene expression of the GABA‐A receptor pathway appears to be mediated by damage to the oolemma during ICSI. Microinjection after IVF reduced gene expression, suggesting that the microinjection damages the embryos during early development.

The GABA‐A receptor pathway plays an important role in early embryonic development.^6^, ^7^ In the present study, we focused on the relationship between the GABA‐A receptor signaling pathway and manipulation during ICSI. Microinjection manipulation tended to exert a greater effect on the expression of GABA‐A receptor signaling pathway genes than culture conditions, hyaluronidase digestion, and PVP, although these factors also appeared to exert minor effects.

Several studies have revealed the correlation between human oocyte loss and the incidence of oocyte degeneration following ICSI, which ranges between 5% and 15%.^2^, ^15^ Oocyte loss and degeneration can be due to the oocyte quality, use of the injection needle, or due to the operator's technique. When the human oolemma was penetrated without additional mechanical stimuli, a higher incidence of degeneration in the oocyte accompanied by low oolemma flexibility. As shown in Figure 1B, the activity of ion‐gated and other channels decreased after ICSI, suggesting that discontinuities in the membrane influences the composition of the cortical oolemma, including the composition of membrane receptors and selective ion channels. This may result in selectivity deprivation of the membrane toward ions or other substances, resulting in the increase in oocyte degeneration after ICSI. Moreover, oolemma disruption induced by microinjection causes a rapid Ca^2+^ influx into the cytosol from the extracellular milieu, which is expected to elicit cell membrane recovery, such as exocytosis, patching, endocytosis, and membrane shedding.^16^, ^17^, ^18^

Different techniques have been applied to rupture the oolemma in ICSI. These techniques have significantly different effects on the oocyte fertilization rate. Carrillo et al have confirmed that oolemma rupture induced by the ICSI injection needle dramatically augments the fertilization rate and decreases the rate of oocyte damage. Oolemma rupture by aspiration into the injection needle leads to over a 50% reduction in damage and almost doubles the fertilization rate, as compared with oolemma rupture induced by directly pushing the needle into the oocyte.^12^

Although previous studies have used RNAi knockdown, which alters the expression of genes of the GABA‐A receptor signaling pathway, or inhibition of the GABA‐A pathway using GABA‐A receptor‐specific antagonists to investigate the role of this pathway, future studies are needed to develop a better understanding of this process. It is still unclear whether the reduction in expression induced by microinjection may also affect the cell proliferation of ES and NCS or long‐term embryonic development. Moreover, GABA receptors are categorized into the GABA‐A, ‐B, and ‐C receptor subtypes. GABA‐A controls proliferation by modifying the chromatin in stem cells. Future studies are needed to determine whether GABA‐B or ‐C have different functions in this process. Further analysis of the GABA receptor signaling pathways as well as the effect of their loss on the transcription, splicing, and the epigenetic state of stem cells may provide more details regarding the other functions of this pathway.

As the knockdown of the GABA‐A receptor signaling pathway results in severe defects in embryonic development, elucidating the mechanism by which microinjection regulates the GABA‐A receptor signaling pathway may provide an intriguing link between ICSI manipulation and embryonic development because the link between these two processes requires further study.

## CONFLICT OF INTEREST

The authors declare that they have no competing interests.

## Supporting information

Figure S1

Table S1
