# Supplementary figures and images for: Microinjection manipulation decreases the expression of GABA‐A receptor signaling pathway genes in mouse embryos derived using intracytoplasmic sperm injection
Source: J Clin Lab Anal. 2020 Sep 20;35(1):e23584. doi: 10.1002/jcla.23584 (PMC7843277; doi:10.1002/jcla.23584)

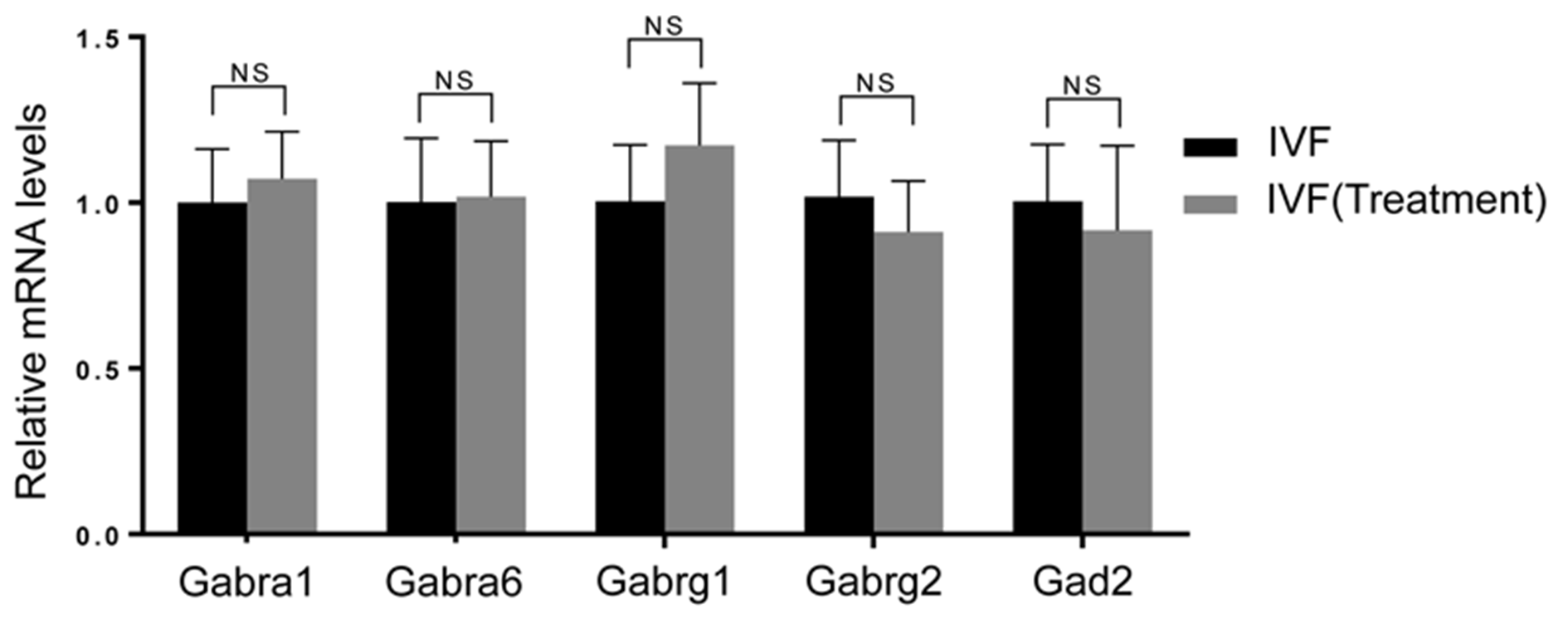

Supplement: Supplementary file 1 — Figure S1 [file JCLA-35-e23584-s001.tif]
