# Supplementary material for: Microinjection manipulation decreases the expression of GABA‐A receptor signaling pathway genes in mouse embryos derived using intracytoplasmic sperm injection
Source: J Clin Lab Anal. 2020 Sep 20;35(1):e23584. doi: 10.1002/jcla.23584 (PMC7843277; doi:10.1002/jcla.23584)
Supplement: Supplementary file 2 — Table S1 [file JCLA-35-e23584-s002.docx]

**Table S1. Comparison of differentially expressed genes involved in the GABA receptor signaling pathway between the IVF and *in vivo* groups from the GSE23009 dataset**

| **IVF vs *in vivo* GABA Receptor Singaling genes** | | |
| --- | --- | --- |
| **SYMBOL** | **logFC** | ***P*.Value** |
| Abat | 0.251599797 | 0.746791 |
| Gabra1 | -0.657917083 | 0.251083 |
| Gabra2 | -0.975446418 | 0.063801 |
| Gabra3 | -0.637335016 | 0.455026 |
| Gabra6 | 0.003467956 | 0.997132 |
| Gabrb1 | 0.760024125 | 0.178965 |
| Gabrb2 | 0.278416656 | 0.326963 |
| Gabrb3 | 0.497468838 | 0.664492 |
| Gabrg1 | -0.254968845 | 0.705854 |
| Gabrg2 | -1.382184295 | 0.020097 |
| Gabrg3 | -1.126413413 | 0.0322 |
| Gabrd | -0.469325334 | 0.137813 |
| Gabre | 0.267489249 | 0.749856 |
| Gabrp | -1.001654209 | 0.295004 |
| Gabrq | -0.190235613 | 0.810138 |
| Gabrr1 | 1.01872931 | 0.16073 |
| Gabrr2 | 0.191647887 | 0.813568 |
| Gabbr1 | -0.075671357 | 0.767752 |
| Gad1 | -0.402028603 | 0.37832 |
| Gad2 | -0.600275998 | 0.312429 |
| Aldh9a1 | 0.667649039 | 0.098726 |
